# Combined Minimally Invasive Surgical and Percutaneous Approaches for a Patient on Hemodialysis With Severe Aortic Stenosis and Complex Coronary Artery Disease

**DOI:** 10.1155/cric/9229849

**Published:** 2025-03-21

**Authors:** Teruaki Wada, Kentaro Honda, Hironori Kitabata, Yoshiharu Nishimura, Atsushi Tanaka

**Affiliations:** ^1^Department of Cardiovascular Medicine, Wakayama Medical University, Wakayama, Japan; ^2^Department of Thoracic and Cardiovascular Surgery, Wakayama Medical University, Wakayama, Japan

**Keywords:** aortic stenosis, coronary artery bypass grafting, minimally invasive cardiac surgery, percutaneous coronary intervention, transcatheter aortic valve implantation

## Abstract

Patients on hemodialysis with concomitant severe aortic stenosis (AS) and multivessel coronary artery disease (CAD) are at high risk for surgical aortic valve replacement (SAVR) and coronary artery bypass grafting (CABG). Transsubclavian–transcatheter aortic valve implantation (TSc-TAVI) is a well-established alternative approach to transfemoral TAVI for patients with unfavorable femoral access. Herein, we report a case in which minimally invasive surgical treatment and TSc-TAVI were performed simultaneously in a patient with severe AS and multivessel CAD undergoing hemodialysis. An 85-year-old man undergoing hemodialysis for end-stage renal disease owing to severe AS (mean pressure gradient, 46 mmHg; aortic valve area, 0.75 cm^2^; and left ventricular ejection fraction, 59%) presented to our hospital with chest pain on exertion. Preoperative coronary angiography revealed significant stenosis of the left anterior descending (LAD) coronary artery and right coronary artery (RCA), requiring revascularization. However, the patient was not a good candidate for transfemoral TAVI because of a porcelain ascending aorta and a shaggy descending aorta observed on computed tomography. He was scheduled for concomitant right TSc-TAVI and minimally invasive cardiac surgery (MICS)–CABG after percutaneous coronary intervention (PCI) for the RCA. The treatment was successful. Simultaneous TSc-TAVI and MICS-CABG with PCI may be applied as a minimally invasive surgical treatment modality for patients with AS and CAD undergoing hemodialysis.

## 1. Introduction

Transcatheter aortic valve implantation (TAVI) is a well-established treatment modality for patients with severe, symptomatic aortic stenosis (AS) [1]. Transsubclavian (TSc)-TAVI is a useful alternative to transfemoral (TF)-TAVI in selected patients with an increased risk of femoral injury [2]. Some patients undergoing hemodialysis for severe AS and concomitant multivessel coronary artery disease (CAD) are not eligible for surgical aortic valve replacement (SAVR) combined with coronary artery bypass grafting (CABG). In such cases, concomitant TAVI and percutaneous coronary intervention (PCI) are considered [3]. If the coronary lesions are suitable for CABG, concomitant TAVI and CABG are required [4]. However, a combined TAVI and CABG approach is more invasive than combined percutaneous approaches (i.e., TAVI + PCI). Regarding simultaneous TAVI with CABG, TAVI with minimally invasive cardiac surgery (MICS)-CABG is another surgical modality for coronary revascularization [5]. Herein, we report a case of TSc-TAVI combined with hybrid MICS-CABG and PCI in a patient on hemodialysis with severe AS and concomitant multivessel CAD.

## 2. Case Presentation

**Figure 1:**
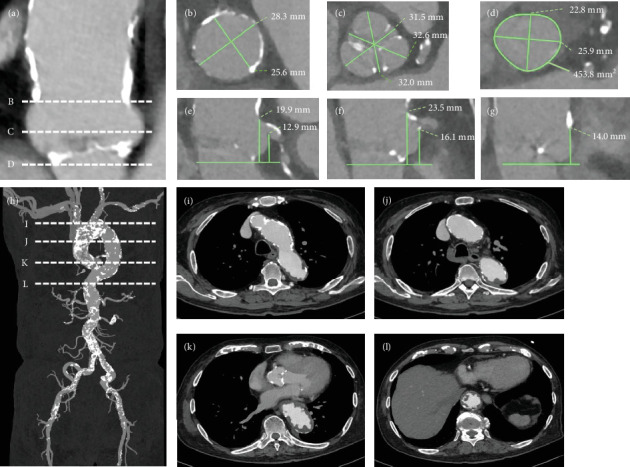
Multidetector CT of the aortic valve and access root. (a) Aortic valve complex, (b) aortic annulus, (c) valsalva, (d) sinotubular junction, (e) left coronary height and cusp height, (f) right coronary height and cusp height, (g) noncoronary cusp height, and (h–l) CT scans showing a shaggy and heavily calcified aorta from the aortic arch to the descending aorta. Abbreviation: CT, computed tomography.

**Figure 2:**
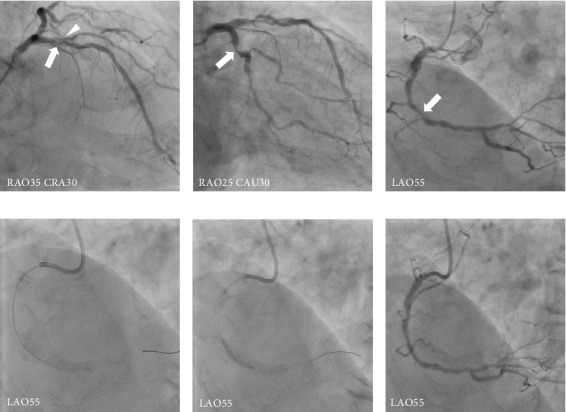
Coronary angiography and percutaneous coronary intervention for the right coronary artery. (a–c) CAG reveals significant stenosis in the proximal LAD (arrow), Dg (arrowhead), and middle RCA (arrow). Intermediate stenosis is observed in the proximal LCX (arrow; FFR: 0.83). (d) OA with a guide extension catheter, (e) angioplasty with a 3.0/10-mm cutting balloon and a 3.0/20-mm drug-coated balloon, and (f) final angiography image. Abbreviations: CAG, coronary angiography; Dg, diagonal branch; FFR, fractional flow reserve; LAD, left anterior descending coronary artery; LCX, left circumflex coronary artery; OA, orbital atherectomy; RCA, right coronary artery.

An 85-year-old man undergoing hemodialysis for severe AS presented to our hospital with chest pain on exertion. The patient's height, weight, and body surface area were 155 cm, 63 kg, and 1.65 m^2^, respectively. A hemodialysis shunt was placed on his right forearm. He was considered to be at high risk for SAVR (Society of Thoracic Surgery score: 8.3%; with a porcelain ascending aorta). Transthoracic echocardiography showed a left ventricular ejection fraction (LVEF) of 59%, a peak aortic valve velocity of 4.5 m/s, a mean pressure gradient of 46 mmHg, an aortic valve area of 0.75 cm^2^, and mild to moderate aortic insufficiency without significant mitral valve disease. Computed tomography (CT) revealed an aortic valve annulus (axis diameter, 22.8 mm × 25.9 mm; area, 454 mm^2^; perimeter, 76.3 mm) (Figures 1a, 1b, 1c, 1d, 1e, 1f, and 1g). His CT scans revealed a porcelain ascending aorta and a shaggy descending aorta (Figures 1h, 1i, 1j, 1k, and 1l); therefore, he was not a good candidate for TF-TAVI. The minimum diameters of the right and left subclavian arteries were 7.5 and 5.5 mm, respectively. We decided to perform TAVI via the right subclavian artery. Coronary angiography findings revealed 90% stenosis in the proximal segment of the left anterior descending (LAD) artery, in the diagonal branch (Dg), and in the middle segment of the right coronary artery (RCA), which indicated revascularization. The proximal segment of the left circumflex coronary artery (LCX) showed 75% stenosis, which was negative for ischemia with a fractional flow reserve (FFR) of 0.83 (Figure 2); thus, the LCX was not indicated for revascularization. The SYNTAX score was 33, and the coronary system was right dominant.

Following a discussion with the heart team, we decided to perform concomitant right TSc-TAVI and MICS-CABG after PCI for the RCA. Initially, PCI was performed on the RCA using a 6-Fr guiding system via the left distal radial artery. Because optical frequency domain imaging (OFDI) (FastView, Terumo Corp) identified severely calcified plaques in the middle RCA, we performed orbital atherectomy (OA) with a 6-Fr guide-extension catheter (GuideLiner, Vascular Solutions) to the target lesion. After OA, using the Diamondback 360 Coronary Orbital Atherectomy System (Cardiovascular Systems Inc.), balloon angioplasty was performed using a 2.5/10-mm cutting balloon for the lesion. Additional angioplasty was performed using a 3.0/10-mm cutting balloon, followed by a 3.0/20-mm drug-coated balloon (Figure 2). The final OFDI revealed a lesion with good expansion and a minimum lumen area of 4.1 mm^2^. One week later, concomitant right TSc-TAVI and MICS-CABG were performed. At first, MICS-CABG was performed using the left internal thoracic artery (LITA)-LAD and LITA-saphenous vein graft (SVG)-Dg. The MICS-CABG incision site was at the fifth intercostal space, based on the cardiac apex level indicated on CT. Subsequently, right TSc-TAVI was performed using a 26-mm Sapien 3 (Edwards Lifesciences, Irvine, California) balloon–expandable transcatheter heart valve (THV) with 3 mL underfilling. The TAVI procedure was successful without major complications. Transesophageal echocardiography showed trivial paravalvular leakage, with no major bleeding noted. After the procedure, the patient was admitted to the intensive care unit (ICU), and continuous hemodiafiltration was initiated.

**Figure 3:**
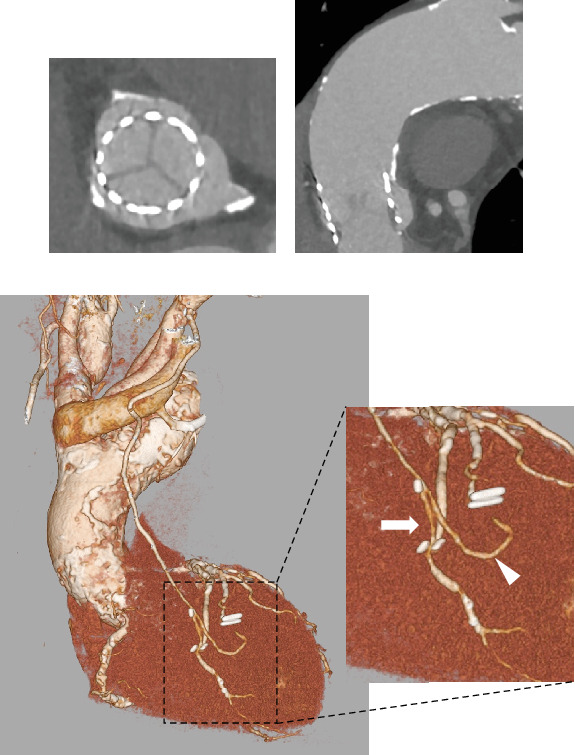
Follow-up coronary computed tomography angiography after transcatheter aortic valve implantation and minimally invasive cardiac surgery–coronary artery bypass grafting. (a) A short-axis CT image of the 26-mm Sapien 3 balloon–expandable THV, (b) a long-axis CT image of the THV, and (c) the LITA-LAD (arrow) and LITA-SVG-Dg (arrowhead) are both patents. Abbreviations: CT, computed tomography; Dg, diagonal branch; LAD, left anterior descending coronary artery; LITA, left internal thoracic artery; SVG, saphenous vein graft; THV, transcatheter heart valve.

The postoperative course was uneventful. On postoperative Day 1, the patient was extubated, and intermittent hemodialysis was initiated. Postoperative transthoracic echocardiography revealed a good THV function (peak aortic valve velocity, 2.0 m/s; mean pressure gradient, 9 mmHg; aortic valve area, 1.62 cm^2^; and trace aortic insufficiency). The patient was discharged from the ICU to the general ward on postoperative Day 4 and discharged home on foot on postoperative Day 17. One year later, the patient had remained angina-free (Canadian Cardiovascular Society (CCS) Class, 0; New York Heart Association (NYHA) Class, I). A follow-up coronary CT angiography showed a good expansion of the THV, patent grafts, and no restenosis of the RCA (Figure 3).

## 3. Discussion

We report a case of severe AS complicated by CAD that was successfully treated with simultaneous TSc-TAVI and MICS-CABG with PCI in a patient undergoing hemodialysis with a high surgical risk.

While sutureless aortic valve replacement has an important role in this patient population [6], it was not an option in this patient because of the porcelain ascending aorta. Older adult patients undergoing hemodialysis for severe AS are at high risk for SAVR, and TAVI is often indicated in such cases. The TSc approach is a necessary treatment modality for patients undergoing hemodialysis with poor arterial accesses, as well as for those not undergoing hemodialysis. Our patient was not a good candidate for a TF approach because of a shaggy descending aorta. Accordingly, we selected the right TSc approach for TAVI to reduce the TAVI catheter travel distance in addition to the fact that we utilized the LITA.

Transcarotid (TC)-TAVI is associated with similar mortality and a significant reduction in stroke compared with the TSc approach [7]. In this patient, we assessed the carotid arteries using both CT and ultrasound; as a result, we determined that the TC-TAVI approach was feasible. However, the TC-TAVI had not been approved at that time in Japan, so the TSc approach was chosen. The minimum diameters of the right and left subclavian arteries were 7.5 and 5.5 mm, respectively. On the other hand, MICS-CABG was performed using the LITA, so we did not use the left subclavian artery for the reason of avoiding vascular complications. Thus, TAVI was performed via the right subclavian artery.

Several studies have reported a correlation between incomplete revascularization (a high residual SYNTAX score) and poorer clinical outcomes, such as increased mortality or major adverse cardiac or cerebrovascular events, supporting PCI in the peri-TAVI period [8–13]. One study reported that the presence of complex CAD in patients undergoing TAVI was associated with significantly poorer 5-year outcomes [14]. No demonstrable difference in outcomes was observed at 1 year in patients who underwent PCI compared with no PCI prior to TAVI in the ACTIVATION trial [15]. This could in part be due to the difference in patient populations, as prior meta-analyses have examined complex CAD with high SYNTAX scores, whereas the ACTIVATION trial cohort mainly exhibited single-vessel CAD. In the NOTION-3 trial, among patients with stable CAD and severe symptomatic AS with an FFR of ≤ 0.80 or a coronary artery diameter stenosis of ≥ 90%, as assessed using angiography, who were undergoing TAVI, revascularization with PCI was associated with a lower risk of a composite of death from any cause, myocardial infarction, or urgent revascularization compared with conservative treatment [16]. Findings from a previous case report and a meta-analysis suggested that using the FFR pre-TAVI might lead to an underestimation of the physiological significance of coronary lesions when using a standard cutoff value of 0.80 [17, 18]. However, the clinical effect of FFR underestimation may be limited because, in patients with untreated CAD, the FFR value crosses the 0.80 cutoff point in approximately 10% of the patients when the FFR pre-TAVI is compared with the FFR measured 6 months post-TAVI. The FFR in patients undergoing TAVI appears to predict outcomes. However, there is no evidence that revascularization of the coronary arteries with an FFR value > 0.80 leads to improved outcomes in patients undergoing TAVI. Given that our patient experienced no angina post-TAVI, we consider it reasonable not to have performed revascularization of the LCX with an FFR of 0.83.

We consider that the cases involving multiple coronary lesions are appropriate for CABG and that concomitant TAVI and CABG are crucial options for revascularization. The strategy for coronary revascularization varies according to the institution and heart team. One study reported that patients on dialysis who underwent off-pump CABG (OPCAB) presented a modestly increased survival compared with those undergoing on-pump CABG [19]. Furthermore, mediastinitis has been associated with increased mortality during the first year post-CABG and a threefold increase during a 4-year follow-up period. Patients with mediastinitis were more likely to present with preoperative dialysis-dependent renal failure [20]. We considered that minimizing the risk of mediastinitis through avoiding a median sternotomy in this dialysis patient granted a major clinical benefit.

Zubarevich et al. [4] reported on high-risk patients who underwent concomitant transaortic TAVI and OPCAB. They concluded that a hybrid approach combining transaortic TAVI and OPCAB may be a safe and feasible treatment modality for high-risk patients who are ineligible for conventional surgical or interventional therapies. Baquero et al. [21] also reported that OPCAB and transaortic TAVI were effective, even in the presence of a porcelain aorta. Moreover, Pirelli et al. [5] reported that staged TAVI after hybrid minimally invasive direct coronary artery bypass surgery was effective for combined complex CAD and AS.

In this patient, we adopted a simultaneous MICS-CABG and TAVI strategy. In this hybrid strategy, we achieved a less invasive approach for this elderly dialysis patient than the two-staged strategy, in which MICS-CABG was performed after TAVI. We have reported on TAVI with MICS-CABG as a novel treatment modality for older adult patients with combined AS and complex CAD with a high surgical risk [22]. A single-staged MICS-CABG and TAVI strategy could improve clinical outcomes after surgery, compared with conventional concomitant OPCAB and TF-TAVI. Because the coronary lesions of the LAD and Dg were complex for bifurcation, PCI was at high risk for periprocedural myocardial infarction owing to the occlusion of the Dg. Therefore, we considered CABG preferable to PCI as the revascularization method. If the coronary lesions are limited to the left coronary artery (LCA), MICS-CABG would likely be the best treatment option among CABGs. Because our patient had an RCA lesion in addition to LCA lesions, MICS-CABG alone could not achieve complete revascularization.

The current guidelines recommend that PCI before TAVI should be performed in patients with severe CAD (coronary artery diameter stenosis > 70%) only in proximal segments, particularly if presenting with an acute coronary syndrome, symptoms of angina pectoris, or subocclusive lesions (> 90% diameter stenosis) [23]. PCI after TAVI might be associated with improved outcomes compared with PCI before or concomitantly with TAVI in the REVASC-TAVI registry [24]. However, these results should be confirmed in the ongoing randomized controlled trials, such as the PRO-TAVI trial [25]. In this patient, the stenosis of the RCA was located in the middle segment. However, the severity of RCA stenosis was 90% on the right dominant coronary system. Therefore, we decided to perform revascularization of the RCA before TAVI after the heart-team discussion. Subsequently, MICS-CABG was performed for the LAD and Dg (LITA-LAD and LITA-SVG-Dg, respectively).

## 4. Conclusion

We report a case of successful concomitant TSc-TAVI and MICS-CABG with PCI for the treatment of a patient with a shaggy aorta and complex CAD. This minimally invasive treatment may be an effective option for patients with AS and CAD.

## Data Availability

The datasets analyzed in this case report are available from the corresponding author upon reasonable request.
